# Comparative Analysis of Diurnal Thermal Stress Responses and Lag Effects in *Acer Campestre* Using Chlorophyll Fluorescence During UK Summers 2022–2023

**DOI:** 10.1007/s41748-025-01021-2

**Published:** 2026-01-20

**Authors:** Ramla Khan, Philip Wheeler, David Gowing

**Affiliations:** 1 School of Earth, Environment and Ecosystem Sciences, The Open University, Milton Keynes, UK; 2 Nuffield Department of Orthopaedics, Rheumatology and Musculoskeletal Sciences, The University of Oxford, Oxford, UK

**Keywords:** Ecophysiology, NPQ, ETR_max_, Time-lag effects, Generalised linear mixed models (GLMMs), Heat stress, Urban forestry

## Abstract

Urban trees face increasing extreme heat, yet the timing of their short-term physiological responses remains unclear. This study investigated thermal stress dynamics in *Acer campestre* during two contrasting UK summers (2022 and 2023) and examined how sampling frequency affects the detection of temporal lag effects. Chlorophyll fluorescence parameters (NPQ and ETR_max_) were measured daily during the 2022 high heat days and weekly under the milder conditions of 2023. Generalised linear mixed models and cross-correlation analyses explored the relationships between leaf temperature and photosynthetic performance. In 2022, NPQ showed increased morning thermal sensitivity under extreme heat, but detrended analyses did not reveal strong multi-day lag effects. ETR_max_ responded in synchrony with temperature, showing no detectable time lag. In 2023, apparent multi-day correlations matched exactly with the weekly sampling interval and were diminished or nullified by detrending, suggesting sampling artefacts rather than physiological memory. Across both years, morning measurements consistently indicated the greatest thermal sensitivity. These findings reveal that *Acer campestre* exhibits threshold-dependent stress responses and highlight the importance of high-frequency sampling for accurately capturing short-term physiological dynamics. Sparse sampling can produce misleading lag patterns, emphasising the need to match measurement frequency with the biological response timescales in urban tree monitoring.

**Graphical Abstract:**

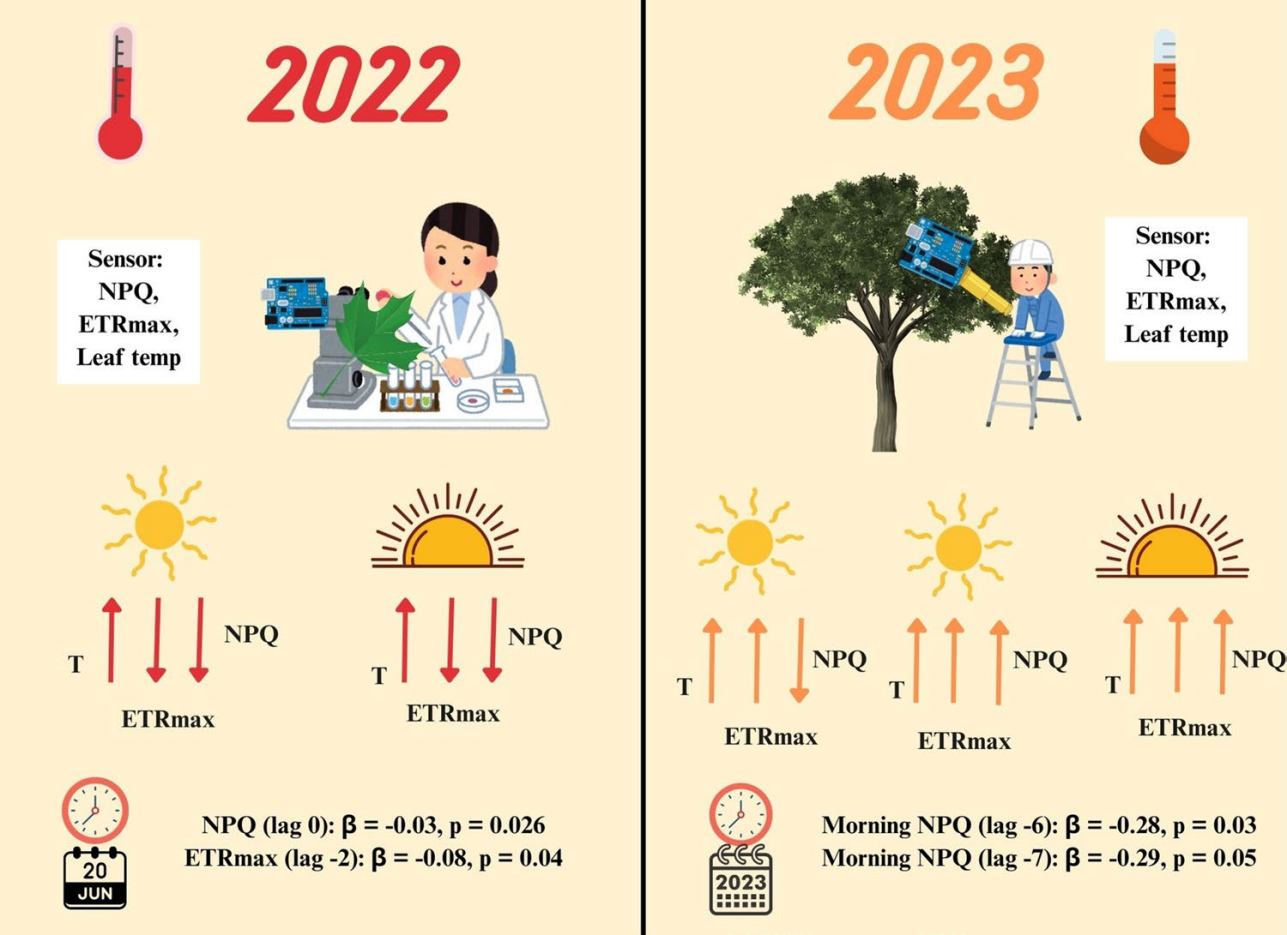

This graphical abstract describes the contrasting methodological approaches and key physiological findings between the two years. The study compared extreme heat conditions in 2022 (depicted by the red thermometer and text) with moderate thermal conditions in 2023 (orange thermometer and text). The left panel displays laboratory measurements from 2022, where a researcher in lab attire collected detached leaf samples for chlorophyll fluorescence analysis using specialised sensors measuring NPQ, ETR_max,_ and leaf temperature (T). The right panel shows field-based canopy measurements from 2023, featuring a researcher with safety equipment using a ladder to reach tree canopies with the same sensor technology. Diurnal response patterns are illustrated through sun symbols representing morning, midday, and evening measurements, with directional arrows indicating parameter trends. In 2022, extreme heat caused declining NPQ and ETR_max_ responses (downward red arrows) despite rising temperatures (upward arrows), indicating physiological stress. The year 2023 showed mixed responses with NPQ decreasing in the morning but increasing later, while ETR_max_ consistently increased throughout the day (upward orange arrows), suggesting normal diurnal optimisation without thermal limitation. The temporal analysis results are summarised at the bottom, with clock and calendar symbols representing lag effects and sampling frequency differences, respectively. Daily sampling in 2022 revealed morning thermal sensitivity under extreme heat, NPQ showing strong morning responsiveness (β = -0.03, *p* = 0.026) and ETR_max_ responding synchronously to temperature with no detectable lag (β = -0.08, *p* = 0.04). Weekly sampling in 2023 detected morning-specific correlations at sample intervals − 6 and − 7 (β = -0.28 and − 0.29, respectively), which represent sampling structure artefacts rather than biological memory, highlighting the critical importance of sampling methodology in detecting genuine temporal lag effects in plant stress responses.

## Highlights

Acer campestre shows contrasting heat stress responses across two UK summers.

Heat and sampling frequency strongly influence detection of physiological lags.

Morning-specific NPQ sensitivity observed in both years, strongest in 2022.

Sparse sampling in 2023 produced interval-based correlations, not true memory.

Heat intensity and sampling frequency critically shape physiological lag detection.

## Introduction

Urban trees play a key role in meeting the United Nations’ Sustainable Development Goals (SDGs) by reducing the urban heat island effect and regulating microclimates, which improves thermal comfort (Armson et al. 2012; Coutts et al. 2016; Kaplan 1984). However, despite their recognised role in heat-mitigation strategies, the short-term physiological dynamics that determine how urban trees respond to extreme heat remain poorly understood, leading to significant environmental, economic, and public health crises (Calvin et al. 2023).

Recent climatological analyses emphasise that extreme heat events are intensifying globally. For example, Khan et al. (2025a, b) observed an increase in Wet Bulb Globe Temperature (WBGT) across the Arabian Peninsula over the past five decades, indicating rising cumulative heat exposure. Similarly, Khan et al. (2025a, b) demonstrated significant shifts in potential evapotranspiration (ETₚ) trends, reflecting changes in atmospheric evaporative demand under climate change. However, these climatological indices describe atmospheric or human heat exposure, not plant physiological stress. Plants experience heat primarily through leaf temperature, transpiration, and leaf-energy balance, which can diverge substantially from air temperature under high radiation or limited evaporative cooling. This distinction is critical because leaf-level heat stress directly impairs photosynthetic efficiency and disrupts energy dissipation pathways (Martínez-Villa et al. 2024). Heat stress also damages core components of the photosynthetic apparatus, with chlorophyll fluorescence proving highly effective for diagnosing these effects (Zhao et al. 2020). Repeated or intense heat waves can further alter thermal tolerance thresholds and exacerbate damage to photosystems, affecting leaf metabolism and stress acclimation processes (Ahrens et al. 2021). Against this background of increasing climatic heat exposure and plant physiological vulnerability, understanding the timing and persistence of leaf-level responses is essential. Chlorophyll fluorescence parameters provide a sensitive and rapid means of detecting heat-induced stress, making them well-suited for evaluating short-term lag effects and diurnal variation in tree physiological performance under contrasting thermal conditions.

Rising temperatures pose a substantial threat to urban tree health by disrupting key physiological processes such as photosynthesis. Heat stress reduces photosynthetic efficiency, slowing down carbon assimilation and limiting tree growth and survival (Ahrens et al. 2021; Sharkey 2005). Research shows that by 2099, 82 out of 140 urban tree species currently thriving in California would be rendered unfit for planting due to the region’s expected temperature increases (McBride and Laćan 2018). Similarly, studies in Europe have shown that certain tree species, such as *Fagus sylvatica*, are already experiencing stunted growth in southern regions due to rising temperatures (Bosela et al. 2016).

The effects of elevated temperatures on trees at the leaf scale can be assessed with parameters derived from the light response curve through chlorophyll fluorescence methods (Benedetti et al. 2018; Feeley et al. 2020). These parameters provide valuable insights into how efficiently plants convert absorbed light into energy and dissipate excess energy under stress. Key indicators include non-photochemical quenching (NPQ), a process in which excess absorbed light energy is dissipated as heat to protect plants from photodamage (Li et al. 2015; Ruban 2016) and Electron transport rate (ETR_max_), which indicates photosynthetic capacity and efficiency, with higher values showing better stress resilience (Baker 2008; Shanker et al. 2022). Light response curve fitting also yields the initial slope (α, dimensionless, representing light-use efficiency at low light) and the convexity parameter (θ, dimensionless, describing the curvature of the transition from light-limited to light-saturated photosynthesis (Peri et al. 2005).

A growing body of research suggests that trees exhibit delayed physiological responses to heat stress, where recovery extends well beyond the actual heat event (Rijnhart et al. 2022). These temporal dynamics range from immediate responses like stomatal conductance changes to longer-term adjustments in photosynthetic capacity, also known as time lag effects, taking days or weeks (Sage and Kubien 2007). Zhu et al. (2024) demonstrated that lag response times vary by specific physiological process, while showing that these responses interact with plants’ circadian rhythms. Understanding these time-lagged effects is essential for predicting tree performance under increasingly variable climate conditions, particularly in urban environments where heat stress events are becoming more frequent and intense.

Despite increasing research on heat stress on trees, key gaps remain in understanding how these delayed responses manifest in urban settings. While previous studies have examined tree responses to heat across various species and contexts, much of the existing work has been conducted in natural forests or agricultural settings, with limited studies examining the physiological resilience of trees specifically in urban microclimates. Moreover, many of these studies do not consider the lagged response between variables.

We hypothesise that a common urban area tree found in the UK exhibits time-lagged physiological responses to thermal stress, with the magnitude and timing of these responses varying diurnally and seasonally. The study objectives are: to quantify temporal lag effects in NPQ and ETR_max_ responses to leaf temperature, to compare diurnal patterns between morning and evening measurements, and to assess how these responses differ between years of contrasting thermal intensity.

## Study Species

*Acer campestre* (field maple), belonging to the Sapindaceae family, is a widely planted native species in the UK and common in urban environments. It was selected for this study because it is one of the most prevalent urban tree species in UK streetscapes and managed landscapes, making it a relevant model for understanding heat-stress responses in local urban forests. Its moderate heat tolerance, compact canopy architecture, and well-characterised chlorophyll fluorescence behaviour make it suitable for physiological monitoring and ensure that responses can be interpreted within an established ecophysiological framework (San-Miguel-Ayanz et al. 2016).

We purchased the field maple trees in this study from Barcham Trees, Nursery in Soham, Cambridgeshire, England. They specialise in container trees and have been providing their services for thirty-five years. In 2024, they were awarded a royal warrant as specialist container tree growers by King Charles III of the UK.

**Fig. 1:**
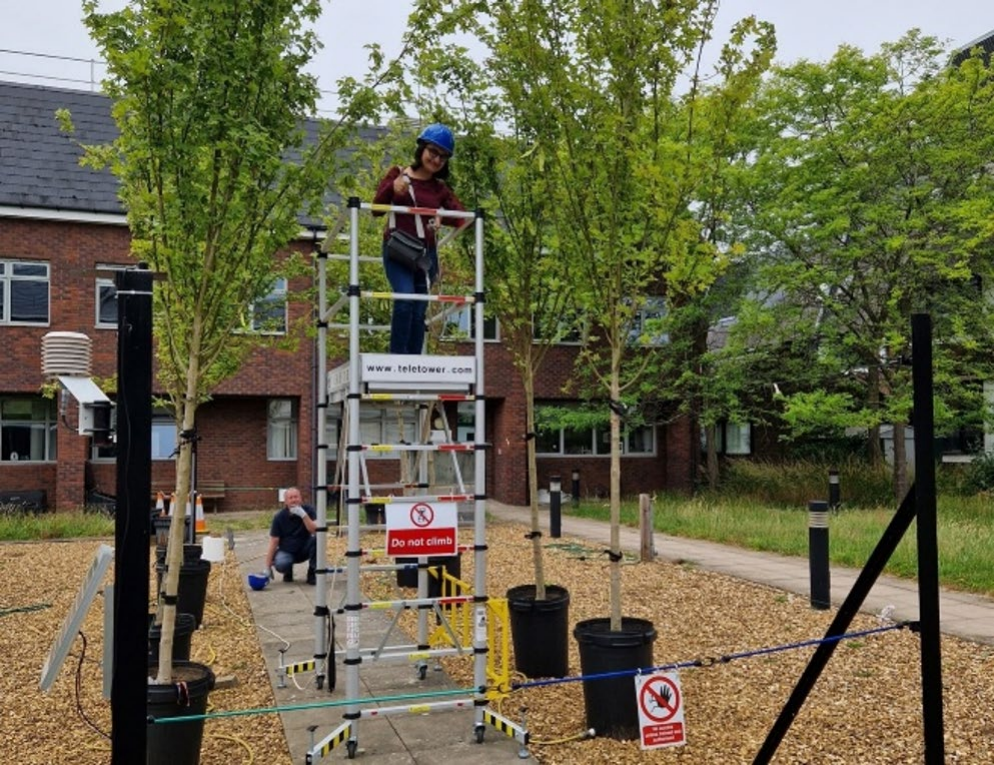
Researchers are using a tele-tower for data collection. The area was closed to the public by roping off and adding proper warning signs

We purchased field maple trees from Barcham Trees Nursery in Soham, Cambridgeshire, England, a specialist container tree grower awarded a royal warrant by King Charles III in 2024. The trees had trunk diameters of approximately 8–10 cm (± 2 cm). The middle of the canopy was accessed using a 2 m high tele-tower with a platform width of 0.6 m and length of 1.4 m (Fig. 1).

## Methodology

In this study, we used two different measurement approaches: detached-leaf sampling in 2022 and on-tree canopy monitoring in 2023, both applied to the same trees under similar conditions across two seasons. In 2022, the controlled conditions inside a laboratory with consistent temperature allowed us to establish baseline measurements for key traits and temperature responses without environmental interference. The transition to field measurements in 2023 added ecological validity by capturing how these physiological processes operate under natural conditions, including fluctuations at different times of day. This approach bridged controlled experimentation with real-world application. All leaves were selected from the upper, sun-exposed portion of the canopy to maintain consistency in light history and thermal exposure.

The shift from daily measurements in 2022 to weekly canopy-based measurements in 2023 reflected two distinct but complementary objectives of the study. The exceptional heatwave conditions of 2022 provided a unique opportunity to capture high-resolution temporal dynamics of physiological stress responses during extreme thermal events. Daily sampling was therefore selected to ensure that short-term lag effects could be resolved under these conditions.

In contrast, the 2023 season was characterised by milder July and August temperatures and was designed to evaluate whether lower-frequency sampling typical of practical field monitoring in urban forestry can detect comparable lag patterns. Weekly canopy measurements represent a realistic ecological sampling interval, especially when access equipment and dark-adaptation requirements constrain continuous high-frequency measurements.

By deliberately employing two contrasting sampling resolutions across years of differing thermal intensity, this study assesses not only the physiological responses to heat stress but also how sampling frequency shapes the detectability of true versus artefactual lag effects. 

### Experimental Setup

**Fig. 2:**
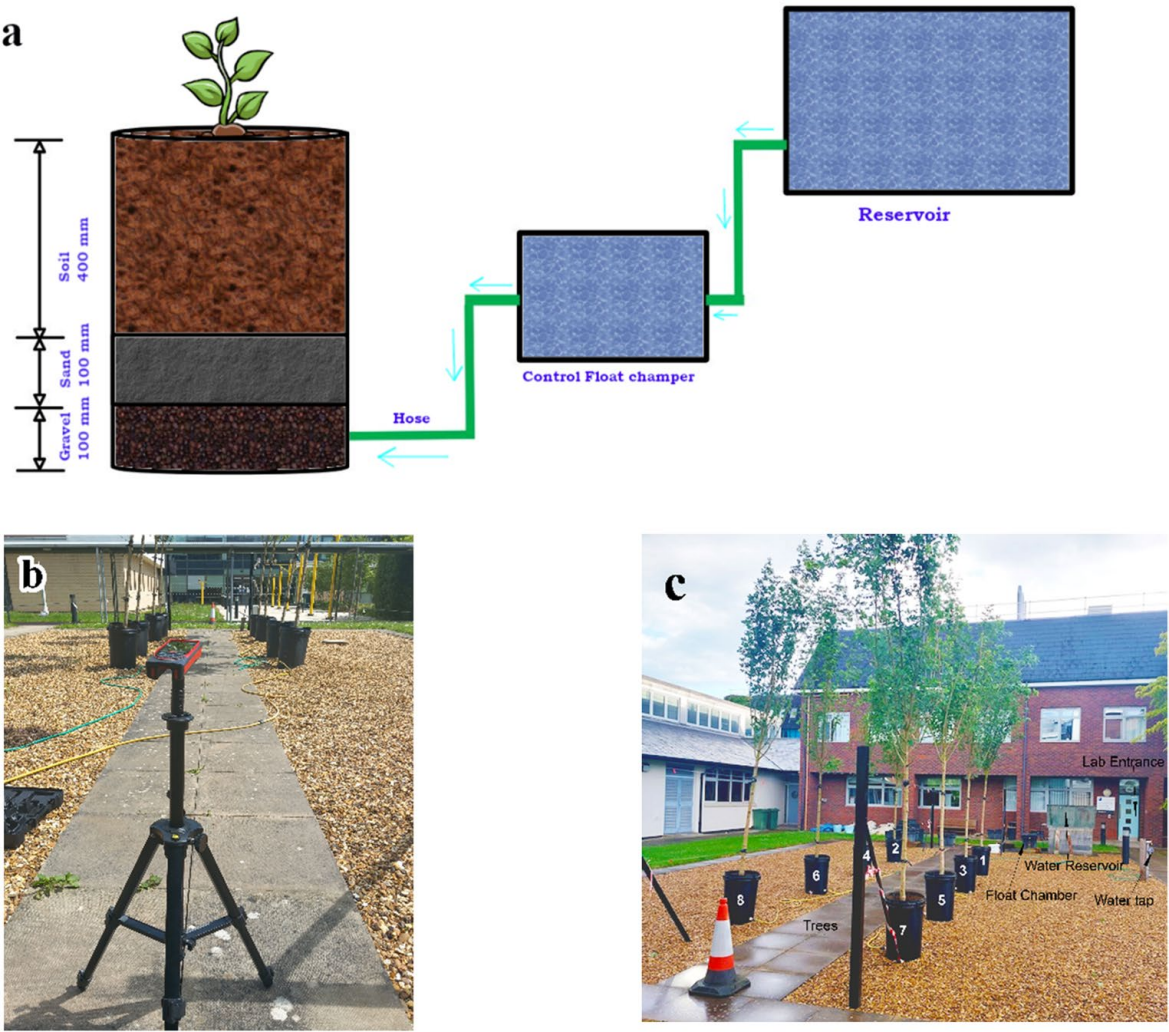
Experimental setup;** a**) Controlled water depth system, **b**) Laser leveller for accurate placement of pots, **c**) Numbered trees with labelled water reservoir and float chambers near the marked lab entrance

Eight field maple trees were placed in an array of 90-litre pots connected to an irrigation system based on the experiment by Araya et al. (2010). The system was engineered to maintain consistent soil moisture conditions and eliminate water stress as a confounding variable in thermal stress assessment. Each 90-litre pot was constructed with stratified layers to ensure optimal drainage and root development. The bottom layer consisted of coarse gravel (5–10 mm diameter, 15 cm depth) for drainage, followed by a middle layer of fine sand (0.5–2 mm diameter, 10 cm depth) for water retention and filtration, and a top layer of potting soil (55 cm depth) mixed with field soil for root establishment. A geotextile weed membrane separated each layer to prevent substrate mixing while allowing water movement (Fig. 2a). All pots were positioned using a laser level to ensure uniform water distribution across the array (Fig. 2b).

The mesocosm system was established in May 2022, with trees acclimated for four weeks before data collection commenced in June. The same trees remained in the mesocosm system through winter and were used for weekly field measurements throughout the 2023 summer season from June to August.

The automated irrigation system maintained a constant water table through capillary action using 12 mm diameter hoses that connected each pot to a central water distribution chamber. Small water chambers (5-litre capacity) equipped with float valves maintained consistent water levels in each pot, while a 200-litre main water tank provided a continuous supply to the distribution system. Daily visual checks of water levels and twice-weekly refilling of the main reservoir were regularly conducted. Each tree was secured to a horizontal galvanised steel cable (6 mm diameter) stretched between support posts to prevent wind damage to ensure uniform water distribution across the array. The pots were arranged in a 2 × 4 grid with 1.5 m spacing between centres to minimise mutual shading, and the entire mesocosm system was positioned in an open area adjacent to the laboratory facility with full sun exposure and natural wind flow (Fig. 2c). The water table was maintained at 50 cm below the soil surface inside the pots to ensure adequate moisture without waterlogging.

The mesocosm setup was equipped with continuous environmental monitoring, including a weather station on the roof of the lab, approximately 2 m above the canopy that recorded air temperature, solar radiation, wind speed, and humidity measurements at hourly intervals.

This controlled mesocosm system enabled isolation of temperature stress effects while maintaining ecologically relevant growing conditions, providing a bridge between laboratory-controlled and field-based measurements.

### Microclimatic Conditions

Throughout the experimental period, trees were grown outdoors on the Open University campus outside the EGL laboratories. Microclimatic conditions were recorded by an automated weather station located on the laboratory roof.

In 2022, daily maximum air temperatures ranged from 13.9 °C to 39.9 °C. The mean daily relative humidity was 53.6%, with instantaneous values ranging from 16% to 92%. Mean global solar radiation intensity was 23.2 W m⁻², and peak midday values reached 38.7 W m⁻².

In 2023, daily maximum air temperatures ranged from 15.4 °C to 33.8 °C. The mean daily relative humidity was 74.7%, with instantaneous values ranging from 26.7% to 100% (raw records occasionally exceeded 100%, which were treated as sensor artefacts). No pyranometer data were available in 2023 due to sensor failure; however, a light sensor recorded mean daily values of 976.4 and peak values of 1460.8 (sensor units), which are not directly comparable to global solar radiation.

### Data Collection Protocol for 2022

In 2022, daily measurements were taken from June to August on days when no rain was forecast, and air temperatures exceeded 24 °C at 10:00 am and 04:00 PM, using the OSp5 + modulated chlorophyll fluorometer. On the remaining days, data was collected every three days. Because these periods showed minimal thermal variability and no major physiological fluctuations, missing daily values were interpolated using linear interpolation to produce a continuous daily time series for cross-correlation analysis. This approach allowed the temperature-response alignment required for lag detection while avoiding distortion of physiological patterns, as interpolated days occurred only during thermally stable periods. Measurements taken every three days were included in the lag analysis only after linear interpolation to produce a continuous daily series; these interpolated values occurred exclusively during thermally stable periods and therefore did not influence heat-event lag detection.

**Fig. 3:**
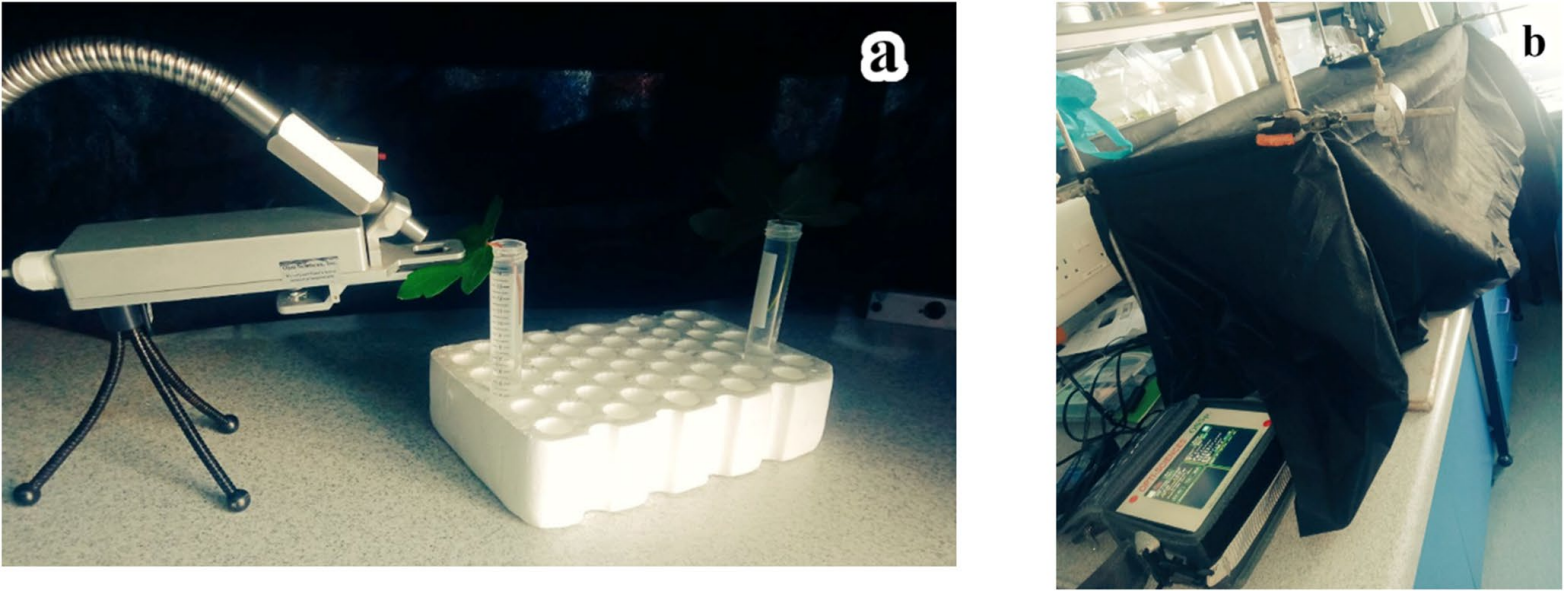
Dark-adapted setup for collecting 2022 fluorescence data using OSp5 + fluorometer: **a**) Leaf sample clamped in PAR clip of apparatus to measure PAR, ETR, and leaf temperature, **b**) Dark cloth clamped on four stands for taking measurements

The trees were positioned just outside the lab, approximately 2 to 3 m from the equipment inside. Leaf samples, detached with pole loppers, were immediately placed in sealed bags, and the stems’ bottoms were cut underwater within the lab to preserve the transpiration bubble and prevent air bubbles from entering the xylem. These leaves were kept in water-filled centrifuge tubes during testing (Fig. 3a). For each leaf, dark-adapted fluorescence readings were first taken as a baseline, followed by rapid light curve measurements at PAR levels of 10–180 µmol photons m^− 2^s^− 1^ using the actinic light source of the apparatus inside a dark-adapted setup, with three-minute intervals between readings (Fig. 3b).

The summer of 2022 was exceptionally warm in the UK, with three heat waves and a number of hot days. This year was recorded as the hottest in decades (National Climate Information Centre 2022; Yule et al. 2023). At the end of the season, we collected a total of 30 days’ worth of data and 240 rapid light curves from which we derived the ETR_max_, Alpha, and theta values. The data is available as a supplementary file.

### Data Collection Protocol for 2023

To measure chlorophyll fluorescence in the tree canopy, a tele-tower was assembled with help from lab staff. Setup began the day before and was completed early in the morning on the day of measurement. Dark-adaptation clips were placed on selected leaves 24 h before data collection. These clips blocked the light, allowing the leaf to adjust to darkness, which was important for accurate fluorescence readings.

Measurements started at 9:30 AM. First, dark-adapted readings were taken by sliding open the clip window and placing the OSp5 + modulated chlorophyll fluorometer PAR sensor over the leaf. After this, the clip was removed, and the leaf was exposed to light for one minute. Then, a light-adapted yield measurement was taken from the same spot on the leaf.

Additional yield measurements were taken later in the day at 01:00 PM and 04:30 PM to monitor changes in photosynthetic efficiency over time. The air temperature at the start of June was high that year, while July and August were mostly wet with mild temperatures. Therefore, data was collected once weekly, resulting in a total of ten days’ worth of data by the end of the summer season.

### Statistical Analysis

#### Parameter Extraction

The lm function in R (R Core Team 2025) was used to fit the non-rectangular hyperbola equation (Eq. 1) to PAR vs. ETR data and extract the required parameters (Cannell and Thornley 1998; Mejdová et al. 2021; Xu et al. 2019; Ye et al. 2013).

1 $$ETR = \frac{{\alpha * PAR + ETR\max - \sqrt {\left( {\alpha * PAR + ETR\max } \right)^{2} - 4 * \theta * \alpha * PAR * ETR\max } }}{{2 * \theta }} $$

The α in Eq. 1 represents the initial slope of the curve, which symbolises the photosynthetic efficiency at low light levels. ETR_max_ represents the saturation point in plant photosynthesis. θ is the factor of curvature that accounts for the transition from linear to saturation.

2 $$NPQ = \frac{{Fm - Fm^{\prime}}}{{Fm^{\prime}}} $$

Fm in Eq. 2 is the maximum fluorescence yield in the dark-adapted state, and Fm’ is the maximum fluorescence yield in the light-adapted state (Opti-Sciences 2013).

#### Modelling the Data

Generalised linear mixed models (GLMMs) are statistical models that account for the non-independence of errors among observations that arise due to repeated measures and clustering (Bakkestuen et al. 2009; Fokkema et al. 2021). The data in our study had repeated measurements due to multiple sample populations, hence GLMM was used to model the data. The lme4 package in R (Bates et al. 2015) was used for fitting the generalised linear mixed models to the time series data (Bolker et al. 2009; Wang et al. 2016). For each response variable (NPQ, ETR_max_), we used a Gaussian error distribution with an identity link, as these physiological traits are continuous and approximately normally distributed following standard fluorescence analyses. The full model structure applied to each time series took the form:

3 $${\rm{Respons}}{{\rm{e}}_{i,t}} = {\beta _0} + {\beta _1}{\rm{Tem}}{{\rm{p}}_{t - {\rm{lag}}}} + (1\mid {\rm{Tree\_ID}}) + {\varepsilon _{i,t}}$$

In Eq. 3, $(1\mid {\rm{Tree\_ID}})$ accounts for repeated measurements on the same tree.

For transparency of statistical reporting, each GLMM also include estimates of the random-intercept variance and the residual variance; these values are reported beneath each model table in the Results. Model-fit statistics were inspected as well. DHARMa diagnostics indicated no gross violation of model assumptions.

Time-series relationships between leaf temperature and fluorescence parameters were examined using the cross-correlation function (CCF) in R (R Core Team 2025). The CCF quantifies correlation across positive and negative lags, enabling detection of temporal lead-lag structures (Liu et al. 2016; Masuda et al. 2022). To minimise the risk of spurious correlations arising from shared seasonal trends, we also computed CCFs on detrended time series (using first differencing), following standard time-series procedures.

## Results

Due to differences in sampling intensity and environmental conditions between years, the following results section is presented using analytical approaches appropriate to each dataset’s structure. The results section is broadly divided into the exceptionally warm summer of 2022 (National Climate Information Centre 2022) and a comparatively milder July and August in the summer of 2023 (Kendon et al. 2024). Our data were broad, with repeated measurements; hence, we used GLMM for modelling to account for this. The leaf temperature, ETR, and PAR values were collected using the inbuilt sensor of the OSp5 + modulated chlorophyll fluorometer.

### Season 2022

Measurements from the 2022 season encompassed 240 rapid light curves (RLCs) derived from eight leaves per day across 30 days. From these, we extracted ETR_max_, Alpha, and theta values, while NPQ values were calculated using the light-adapted and dark-adapted measurements taken before actinic light exposure using Eq. 2.

**Fig. 4:**
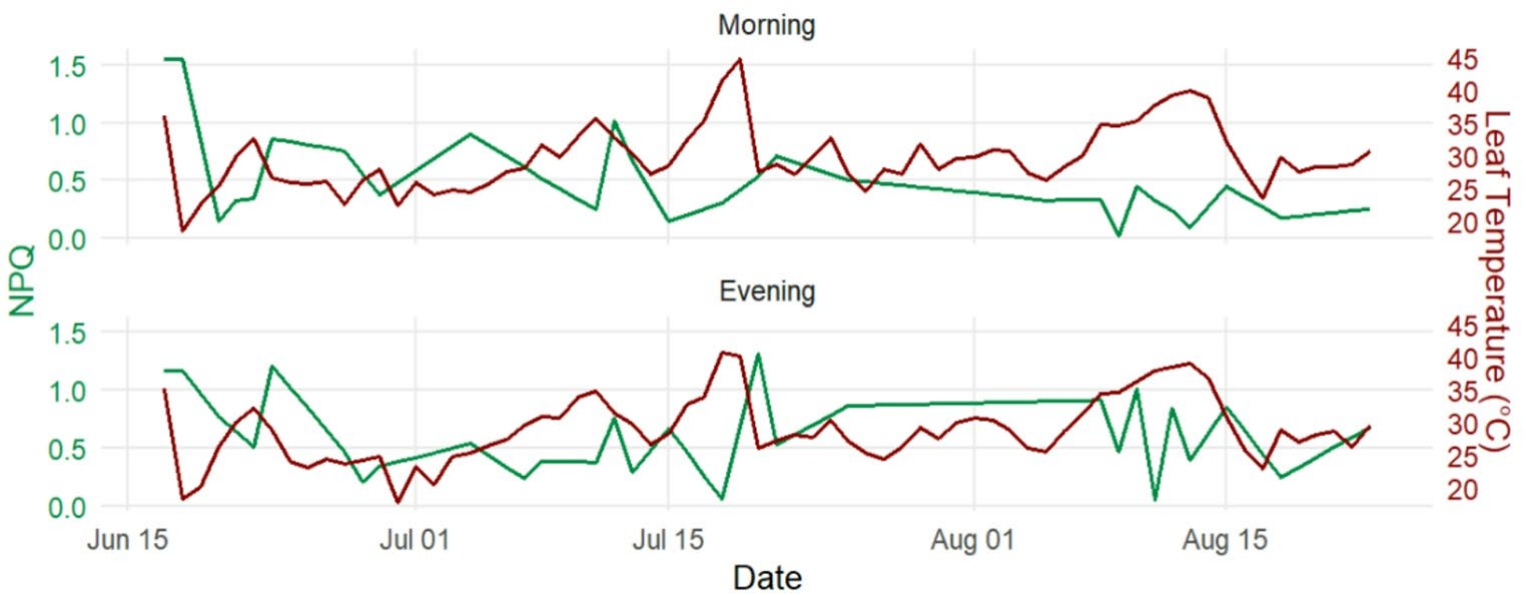
NPQ and Leaf temperature values in 2022 during morning and evening time from June to the end of August. The green lines and axes ticks on the left-hand side of the plot represent NPQ values, while the red lines and axes ticks on the right-hand side of the plot represent leaf temperature values

**Fig. 5:**
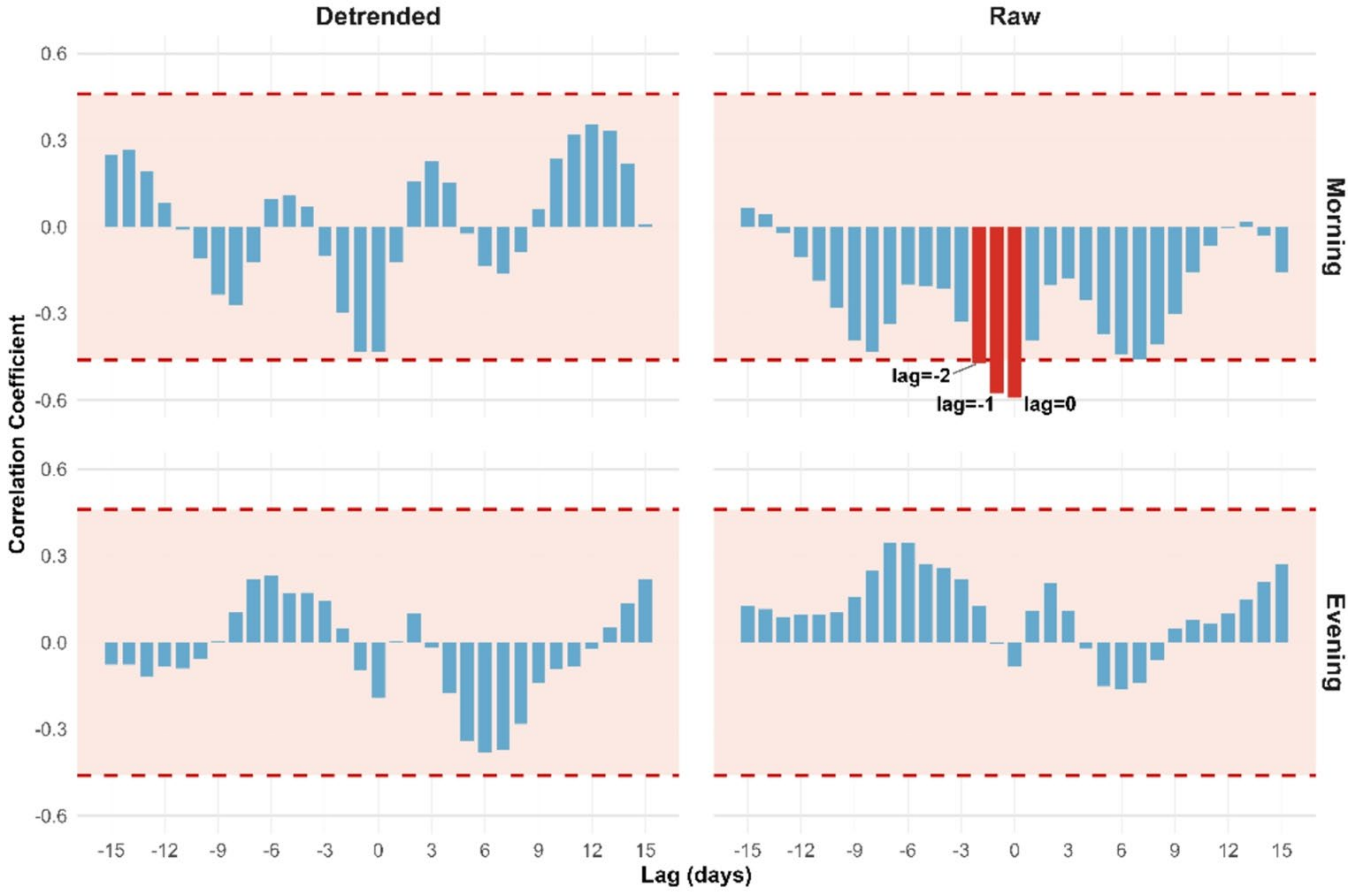
Lag effect between NPQ and leaf temperature in the daily data of 2022 during morning and evening. The blue bars in the plot represent non-significant lags, while the red bars show significant lag days. The right panel shows the result of the raw data, while the left panel shows the results of detrended data

Time series analysis identified a clear temporal delay between NPQ and leaf temperature (Fig. 4). Cross-correlation analysis of the raw data showed that morning NPQ had significant correlations at lags 0, -1, and − 2 days, suggesting strong thermal sensitivity early in the day. Evening measurements showed no significant lag pattern, indicating synchronous responses later in the day. To determine whether these apparent lags resulted from slow underlying trends, we repeated the analysis using detrended (first-differenced) NPQ and leaf-temperature time series (Fig. 5). After removing trends, no lags exceeded the 95% confidence limits. The correlations at lags 0 and − 1 remained near the threshold but were not statistically significant, and the lag at -2 was absent. This pattern suggests that part of the raw lag structure reflected shared low-frequency variation rather than a genuine multi-day physiological delay.

To further investigate this time-dependent relationship, we implemented a generalised mixed-effect model incorporating the observed lag effects for morning data (Table 1). Results revealed that same-day leaf temperature (lag 0) had a statistically significant negative relationship with NPQ (β = -0.03, *p* = 0.02). Specifically, for each unit increase in leaf temperature, NPQ decreased by approximately 0.03 units. Despite the presence of lag effects at -1 and − 2 days in CCF analysis, these lagged temperature effects did not demonstrate statistical significance in the model (lag 1: β = -0.00001, *p* = 0.91; lag 2: β = 0.002, *p* = 0.80). The mixed-effects model included a random intercept for TreeID to account for repeated measurements on individual trees. The random-effects variance was small (TreeID variance = 0.018; SD = 0.134), and the residual variance was 0.022 (SD = 0.148), indicating that most of the variation in NPQ was explained by the fixed effects rather than by between-tree differences. The model showed a good overall fit (log-likelihood = 42.7; AIC = -69.4).

**Table 1:** Output of GLMM model for different lags observed between NPQ and leaf temperature in the morning measurements of 2022

Predictors	(Intercept)	lag 0	lag 1	lag 2
Estimated value	1.18	-0.03	-0.00001	0.002
Std. Error	0.39	0.01	0.01	0.01
p-value	0.007	0.02	0.91	0.80
t-value	3.03	-3.00	-0.001	0.20

**Fig. 6:**
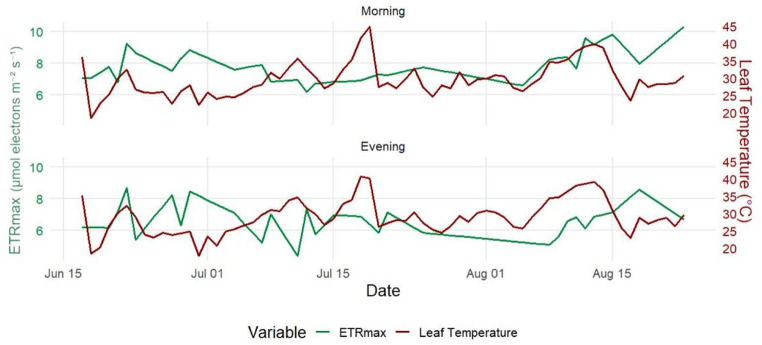
ETR_max_ and Leaf temperature values in 2022 during morning and evening time from June to the end of August. The green lines and axes ticks on the left-hand side of the plot represent ETR_max_ values, while the red lines and axes ticks on the right-hand side of the plot represent leaf temperature values

**Fig. 7:**
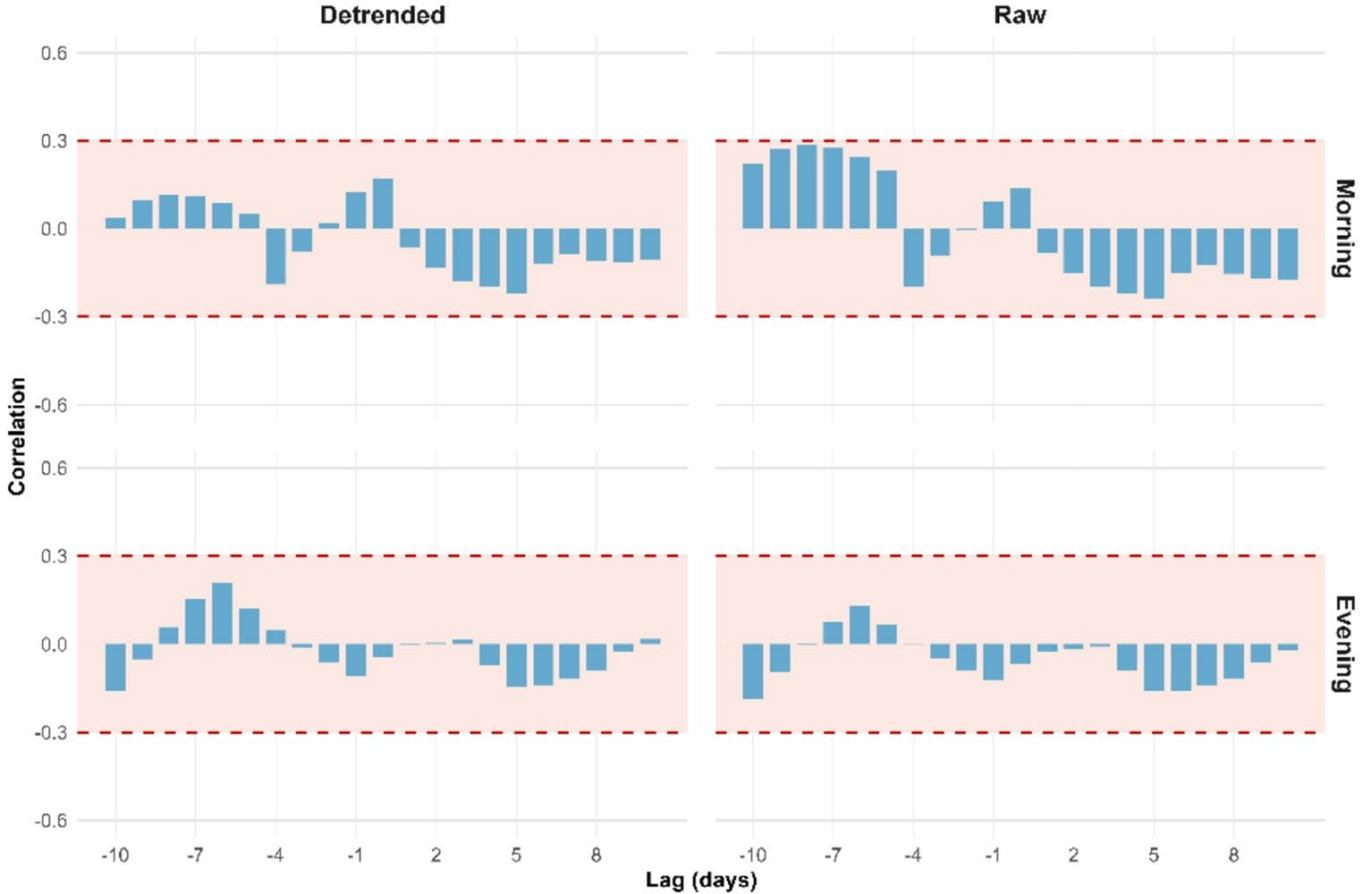
Lag effect between ETR_max_ and leaf temperature in the daily data of 2022 during morning and evening. The blue bars in the plot represent non-significant lags, while the red bars show significant lag days. The right panel shows the result of the raw data, while the left panel shows the results of detrended data

Seasonal variation in ETR_max_ and leaf temperature during morning and evening measurements from June to August 2022 is shown in Fig. 6. In contrast to NPQ, the relationship between ETR_max_ and leaf temperature did not exhibit any clear lag structure in 2022 (Fig. 7). Across all tested lags, neither morning nor evening measurements showed correlations that exceeded the 95% confidence interval. The detrended analysis produced the same outcome, with no significant lagged associations at any time of day. These results indicate that ETR_max_ responded synchronously to temperature during the heatwave period, without evidence of short-term temporal offsets or physiological memory.

### Season 2023

**Fig. 8:**
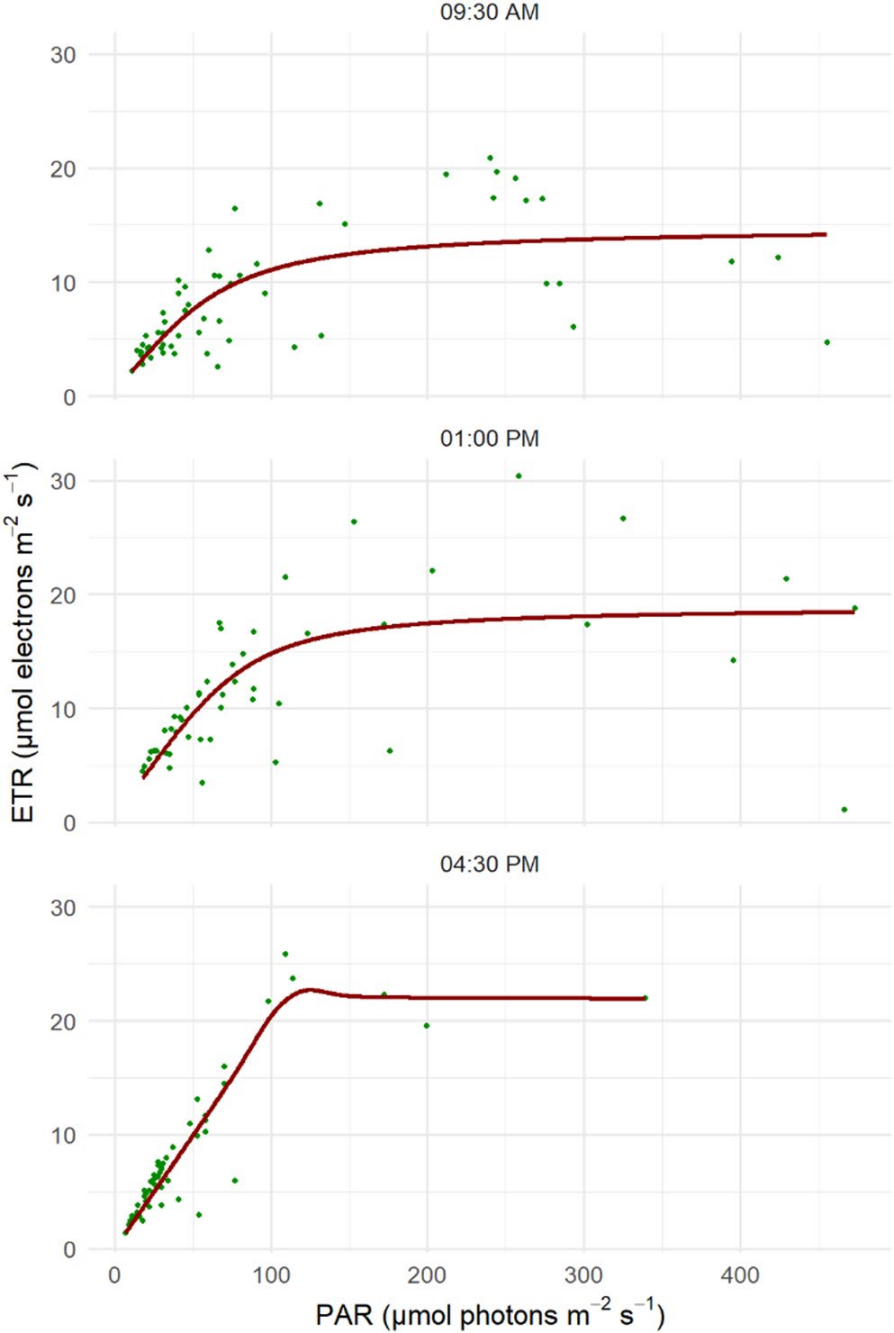
Light response curves for 2023 at different times of day. The green dots represent individual measurements, while the red line shows the fitted curve

Light response curves were fitted using the non-rectangular hyperbola equation (Eq. 1) to the entire season data. Parameters extracted from these curves showed distinct diurnal patterns (Table 2; Fig. 8).

**Table 2:** Physiological parameters derived at different times of day in 2023 using the non-rectangular hyperbola and their corresponding statistical estimations

	Time of Day	09:30 am	01:00 pm	04:30 pm
Alpha	Estimated value	0.19	0.21	0.19
	t_values	3.18	3.84	22.67
	p_values	0.002	< 0.001	< 0.001
	Std. Error	0.06	0.05	0.01
ETR_max_ (µmol m⁻² s⁻¹)	Estimated value	14.84	18.97	21.92
	t_values	6.80	7.33	19.06
	p_values	< 0.001	< 0.001	< 0.001
	Std. Error	2.18	2.59	1.15
Theta	Estimated value	0.74	0.87	1.004
	t_values	1.85	3.87	36.39
	p_values	0.06	< 0.001	< 0.001
	Std. Error	0.40	0.22	0.03

The initial quantum yield (Alpha) exhibited a midday peak of 0.21 at 01:00 pm compared to morning and late afternoon values of 0.19 at 09:30 am and 04:30 pm. This suggested enhanced light utilisation efficiency at midday. All Alpha values were statistically significant (*p* < 0.05), with the strongest significance observed at 04:30 pm (t = 22.67, *p* < 0.001).

Maximum electron transport rate (ETR_max_) demonstrated a progressive increase throughout the day, from 14.84 µmol electrons m⁻² s⁻¹ at 09:30 am to 18.97 µmol electrons m⁻² s⁻¹ at 01:00 pm, reaching a maximum of 21.92 µmol electrons m⁻² s⁻¹ at 04:30 pm. This pattern indicated intensifying photosynthetic capacity as the day advanced. All ETR_max_ values showed high statistical significance (*p* < 0.001), with the highest confidence at 04:30 pm (t = 19.06). ETR_max_.

The convexity parameter (theta) followed an increasing trend from morning (0.74) through midday (0.87) to late afternoon (1.004), suggesting progressively greater light saturation as the day progressed and indicating the transition from light-limited to light-saturated photosynthesis. While midday and afternoon theta values were highly significant (*p* < 0.001), the morning value did not reach statistical significance (t = 1.85, *p* = 0.06).

**Fig. 9:**
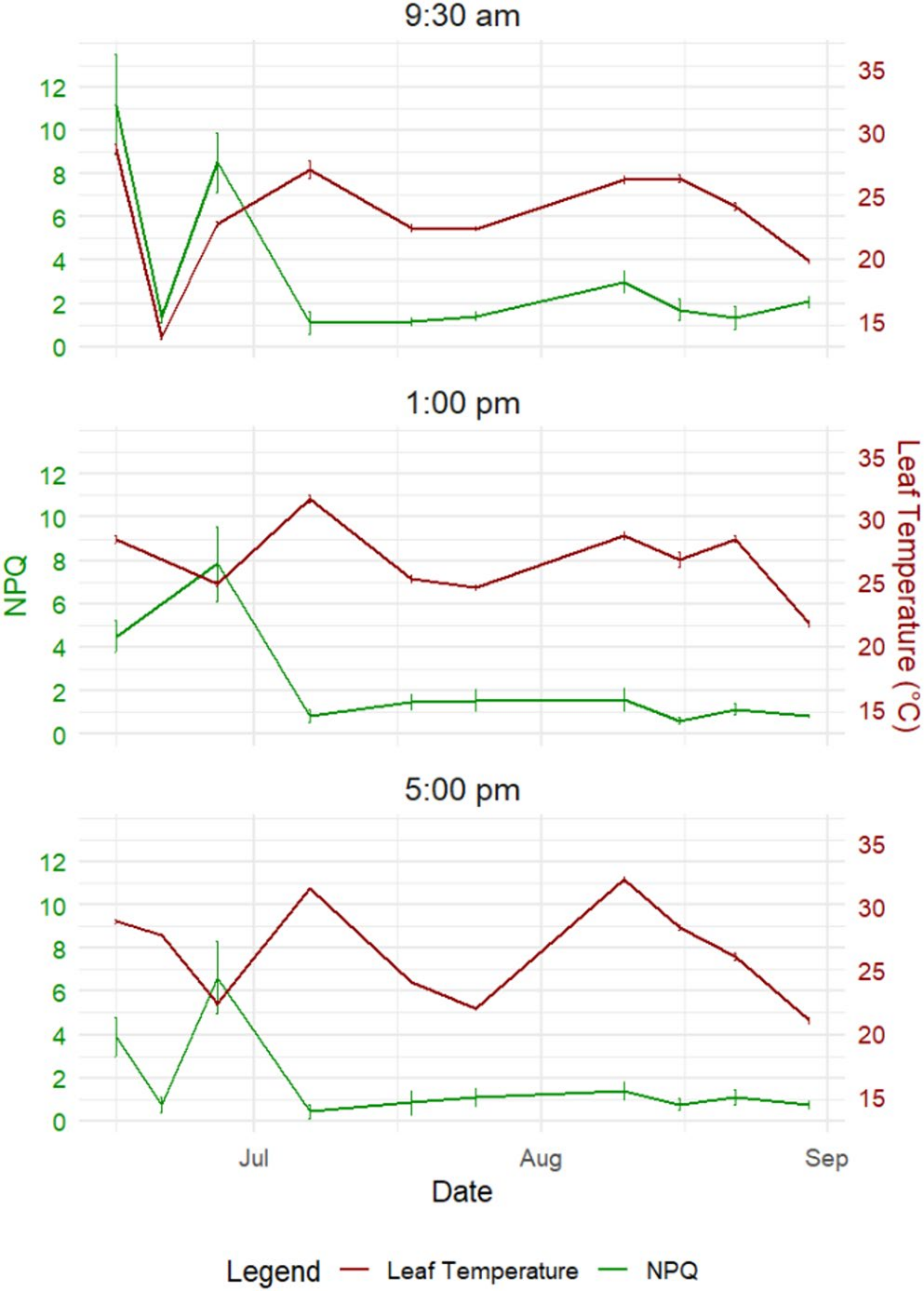
NPQ and Leaf temperature values in 2023 during morning (9:30 am), afternoon (01:00 pm), and evening (05:00 pm) from June to the end of August. The green lines and axes ticks on the left-hand side of the plot represent NPQ values, while the red lines and axes ticks on the right-hand side of the plot represent leaf temperature values

Time series analysis of NPQ throughout the 2023 season revealed elevated values in early July (Fig. 9), indicating increased thermal energy dissipation during this period. To assess temporal relationships between NPQ and leaf temperature, generalised linear mixed-effect models were applied to examine lag effects at different diurnal time points.

**Fig. 10:**
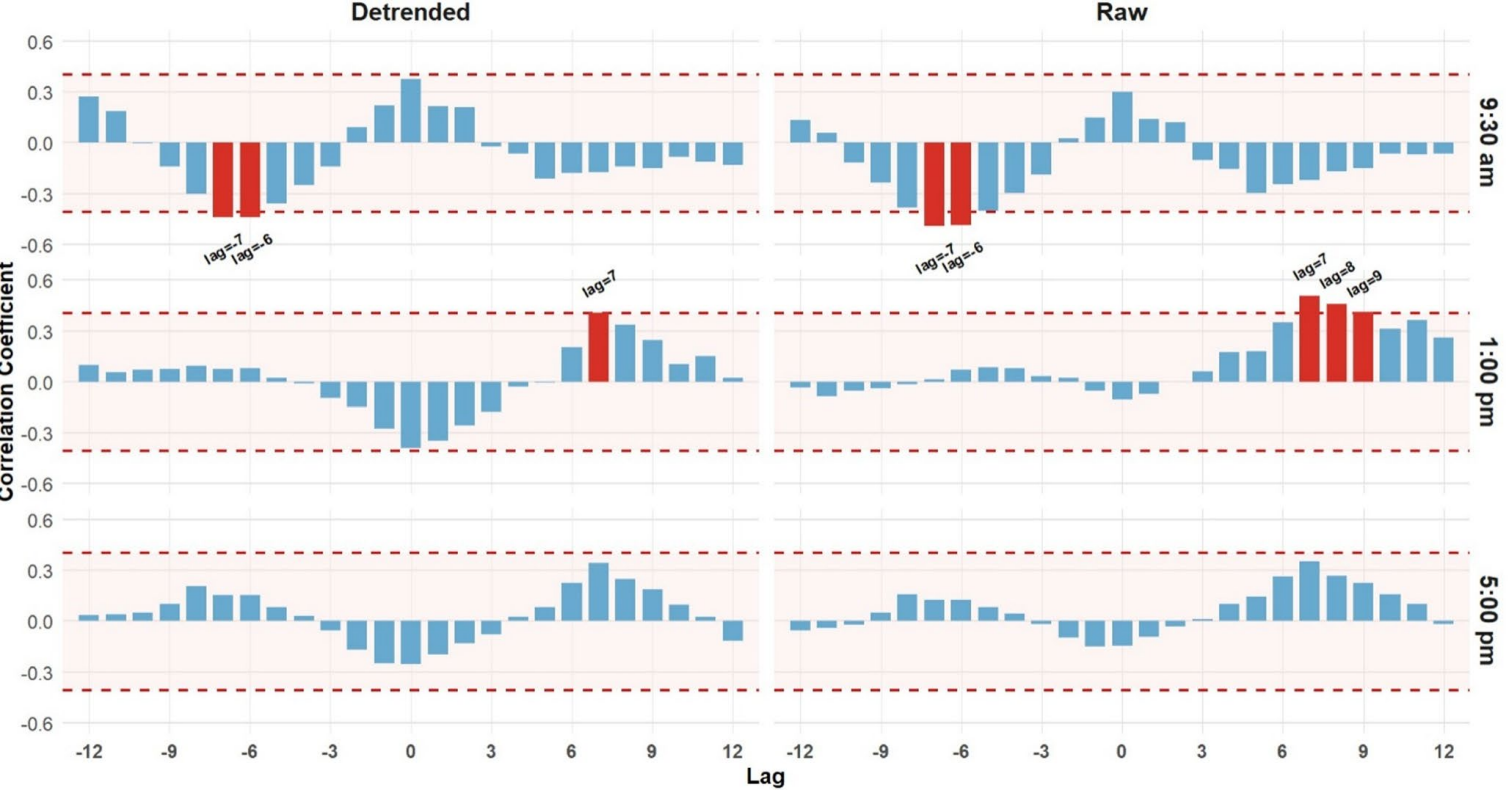
Lag effect between NPQ and leaf temperature in the sparse data of 2023 during morning and evening. The blue bars in the plot represent non-significant lags, while the red bars show significant lags between time points. The right panel shows the result of the raw data, while the left panel shows the results of detrended data

To examine whether the apparent lag structure in 2023 reflected physiological memory or sampling artefacts, we compared raw and detrended CCFs for NPQ across the three daily measurement periods (Fig. 10; Table 3). At 9:30 AM, both raw and detrended analyses showed significant negative correlations at lags − 7 and − 6, corresponding exactly to the weekly sampling interval. At 1:00 PM, the raw data showed significant positive correlations at lags 7, 8, and 9, whereas the detrended analysis retained only the correlation at lag 7. At 5:00 PM, no significant lagged associations were detected in either analysis. Because the same lag positions are preserved after detrending, and because they coincide with the sampling frequency, these correlations arise from the structure of the weekly dataset rather than genuine multi-day physiological memory. Thus, no biologically meaningful lag effects were detected in NPQ during the 2023 season.

**Table 3:** Output of GLMM model for different lags observed between NPQ and leaf temperature in the morning (09:30 am) and afternoon (01:00 pm) measurements of 2023

Time of day	9.30 am		1.00 pm	
lag	-7	-6	7	8
Estimated value	-0.29	-0.28	0.08	0.07
St. Error	0.14	0.13	0.11	0.12
t-value	-2.01	-2.14	0.77	0.58
p-value	0.05	0.03	0.45	0.56

The GLMM for the 2023 NPQ dataset included a random intercept for TreeID to account for repeated measurements of each tree across the ten sampling days. The random-effects variance was minimal (TreeID variance < 0.001; SD < 0.03), indicating very limited between-tree variation in NPQ under the moderate conditions of 2023. The residual variance was 0.017 (SD = 0.130), showing that most variability occurred within trees across time rather than among individuals. The model showed acceptable convergence (log-likelihood = -4.12; AIC = 20.2). Consistent with the CCF analysis, no physiologically meaningful lag structure was detected, and the apparent weekly correlations arose from the sampling interval rather than genuine multi-day memory.

## Discussion

In the United Kingdom, heatwaves in recent years have resulted in thousands of excess deaths, widespread damage to infrastructure and forests, with southern regions facing particularly harsh conditions (Kendon et al. 2021, 2024). Against this backdrop, in this study, we examined the temporal dynamics of heat stress responses in *Acer campestre* using chlorophyll fluorescence parameters (ETR_max_ and NPQ) across two summer seasons (2022 and 2023). We focused on both temporal lag effects and diurnal variation to understand how temperature conditions and thermal history influence physiological responses in this common urban tree species. Our findings revealed that *Acer campestre*’s physiological reactions depend on heat intensity and the time of day, with distinct temporal patterns emerging under different thermal regimes and different data sampling structures, highlighting important implications for tree health monitoring and urban forest management under changing climate conditions.

One of the prominent findings in our study was the presence of different temporal lag patterns between the two seasons, challenging assumptions about consistent stress response timescales in urban trees. During the record-breaking summer temperatures of 2022 in the UK (Dessai et al. 2024; National Climate Information Centre 2022), *Acer campestre* displayed clear morning thermal sensitivity in NPQ during the heatwave period with no evidence of delayed or memory-like behaviour. Under extreme heat conditions, NPQ showed negative associations with leaf temperature at lags of 0, -1, and − 2 days, indicating short-term thermal sensitivity in the raw data; however, detrending removed these lag effects, suggesting that part of the structure reflected shared temporal variation rather than a strict physiological delay (Demmig-Adams and Adams 2006). Although the raw morning correlations suggested short-term responses at lags 0, -1, and − 2 days, these relationships were not retained once slow background variation was removed. Detrending reduced the correlations at lags 0 and − 1 to just below the significance threshold and eliminated the lag at -2 entirely, indicating that part of the apparent lag structure was driven by shared low-frequency dynamics rather than a strict physiological lead-lag sequence. Nevertheless, the proximity of the detrended morning correlations to the confidence limits suggests that NPQ remains more responsive during early-day conditions, even if the temporal offset is less pronounced than implied by the raw dataset. Taken together, these results indicate that NPQ exhibits heightened early-day responsiveness rather than a consistent multi-day lag signal. Unlike NPQ, ETR_max_ did not show evidence of short-term temporal offsets from leaf temperature. Both raw and detrended analyses revealed synchronous behaviour, suggesting that maximum electron transport capacity adjusted directly to current thermal conditions rather than integrating temperature over previous days. This distinction highlights functional differences between photoprotective responses, which can display short-term temporal sensitivity, and photosynthetic electron transport, which in this study showed no detectable lag under extreme heat. Cross-correlation analysis, therefore, indicates that ETR_max_ responded in phase with leaf temperature throughout the 2022 heatwave, with no evidence of delayed or memory-like behaviour.

The negative correlation between NPQ and temperature supports progressive impairment of photoprotective pathways. These lag effects were confined to morning measurements, whereas the absence of evening lags suggests either physiological equilibration after sustained exposure or depletion of protective capacity later in the day. (Li et al. 2024; Zhou et al. 2017). Heat stress progressively damages the D1 protein of PSII, destabilises thylakoid membranes, and suppresses electron transport efficiency, with these changes typically unfolding over 24–48 h owing to transcriptional and translational steps required for repair or downregulation. (Kotak et al. 2007; Murata et al. 2007; Sharkey 2005).

A small portion of the 2022 dataset required interpolation to produce a continuous daily time series during periods when measurements were taken every three days. These intervals occurred exclusively during mild, thermally stable conditions with minimal diurnal variation. Cross-correlation signals observed in 2022 were driven by high-variability heatwave periods that were sampled daily, and sensitivity checks excluding interpolated days showed consistent lag patterns.

One limitation of the 2022 dataset is the use of detached leaves for fluorescence measurements. Leaf detachment can alter stomatal behaviour, transpiration, and leaf temperature, potentially modifying NPQ or ETR_max_ over short time frames. However, detachment effects typically occur within minutes to hours, whereas the lag effects identified here occurred at the scale of 1–2 days. This indicates that detachment may influence absolute values but is unlikely to generate multi-day temporal patterns. Detached-leaf protocols have been widely employed in fluorescence studies under controlled conditions, particularly when consistent light environments are required. Nonetheless, future work using fully non-destructive, canopy-based fluorescence measurements would allow direct comparison of lag dynamics without potential detachment artefacts.

Although detachment inherently changes the leaf microenvironment, our sampling protocol further minimised potential artefacts. Leaves were detached immediately before measurement, placed briefly in sealed bags, and the petiole was recut underwater to maintain the transpiration stream. Measurements were completed within minutes in a laboratory situated only 2–3 m from the experimental trees, limiting any thermal or humidity mismatch. Because detachment-related physiological changes occur rapidly (within minutes to hours), they cannot realistically generate or mask the daily-scale correlations examined in this study. Detachment may influence absolute NPQ or ETR_max_ values, but it is unlikely to introduce or obscure multi-day temporal patterns.

In contrast, 2023 revealed sample interval correlations within a sparse weekly dataset that should not be interpreted as multi-day physiological memory. The negative correlations observed at sample lags − 7 and − 6 in morning NPQ measurements (β = -0.29 and − 0.28, *p* < 0.05) represent statistical associations within the limited 10-day dataset rather than evidence of week-long physiological memory. With only 10 measurement days across the entire season, these lag positions correspond to correlations between NPQ values and temperature measurements from earlier in the same sparse sampling sequence, not genuine multi-day lag effects. The absence of significant correlations at 1:00 PM and 5:00 PM in 2023 supports the interpretation that these effects are related to diurnal timing and morning sensitivity rather than multi-day memory processes. The detrended analysis provides further evidence that the apparent lag structure in 2023 is not physiological in origin. The retention of the same lag positions at − 7 and − 6 in the morning, and at + 7 in the afternoon, demonstrates that these correlations are mathematical artefacts of the weekly sampling interval. No consistent lag structure appeared across times of day, nor did detrending reveal hidden short-term memory effects. These results confirm that under moderate thermal conditions, NPQ varied primarily in response to immediate temperature, and the sparse temporal resolution of the dataset could not resolve the short-term physiological dynamics observed during the 2022 heatwave.

The 2023 season with moderate thermal conditions showed typical diurnal optimisation without short-term lag effects. ETR_max_ increased steadily from morning to late afternoon (14.84 to 21.92, *p* < 0.001), reflecting enhanced photosynthetic capacity without thermal limitation. The light-use efficiency parameter alpha peaked at midday (0.21), indicating optimal quantum efficiency of PSII and efficient light-harvesting complex function during periods of maximal photosynthetic activity (Baker 2008). The progressive increase in theta values throughout the day (0.74 to 1.004) reflects the transition from light-limited to light-saturated photosynthesis, suggesting that photosynthetic machinery becomes increasingly saturated as daily irradiance accumulates (Ye et al. 2013). These patterns demonstrate the tree’s capacity for normal physiological optimisation when not constrained by thermal stress.

The two sampling approaches (detached leaves in controlled laboratory light vs. in situ canopy measurements under natural irradiance) represent different methodological contexts and are therefore treated as separate case studies. The purpose of the comparison is not to directly contrast absolute physiological values between years, but to evaluate how sampling frequency and measurement context shape the detection of lag effects. Differences in irradiance, airflow, and leaf energy balance between laboratory and field settings mean that the two years cannot be interpreted as physiologically equivalent experiments but instead illustrate the methodological influence on time-lag detection.

A key limitation confounding interpretation across these two seasons is the unavoidable link between thermal intensity and sampling frequency. The extreme heatwave conditions of 2022 coincided with high-frequency daily sampling, whereas the milder conditions of 2023 were monitored weekly. The absence of detectable short-term lag effects in 2023 cannot be attributed solely to reduced thermal stress, but rather the sparse sampling resolution inherently limits the ability to detect physiological memory operating on daily scales. Therefore, the two years should not be interpreted as a direct comparative experiment on heat intensity; instead, they represent complementary methodological cases illustrating how sampling frequency interacts with thermal regime to shape whether true lag effects can be detected.

Despite differences in thermal intensity and sampling structure, both seasons demonstrated consistent morning-specific sensitivity. In 2022, this manifested as genuine daily lag effects under extreme heat, while in 2023, it appeared as time-of-day-specific correlations within a sparse dataset under moderate conditions. The consistent finding of morning-specific effects across both years indicates that trees are physiologically most vulnerable during early daylight hours when photosystems are activating after overnight recovery periods. This morning sensitivity may result from circadian clock regulation of stress response genes (Nitschke et al. 2016), lower antioxidant levels following overnight consumption, and rapid transitions from dark-adapted to light-adapted states, increasing photosystem vulnerability (Demmig-Adams and Adams 2006).

These findings have practical implications for urban tree health assessment and climate-adaptation planning. The identification of morning-specific sensitivity and short-term lag effects highlights the importance of timing and temporal resolution in monitoring protocols. Urban heatwaves often coincide with water limitations, compacted soils, and increased evaporative demand; therefore, early detection of physiological decline through high-frequency fluorescence measurements can support proactive irrigation, canopy protection, and species selection decisions. (Chaston et al. 2022; Tan et al. 2016; Wang and Akbari 2016). The demonstration that sparse sampling can generate artefactual lag patterns underscores the need for carefully designed monitoring frameworks, particularly in cities where management actions rely on reliable early-warning indicators of heat stress.

This study’s limitations include the focus on a single species, different sampling frequencies between years that confound temporal interpretation, and relatively small sample sizes. The restriction to leaf-level fluorescence parameters also constrains broader physiological interpretation, as whole-plant responses may involve additional regulatory mechanisms not captured at the leaf scale alone.

Future research should prioritise consistent high-frequency sampling across different thermal conditions to properly distinguish genuine physiological memory from sampling artefacts. Studies should expand to multiple species with varying thermal sensitivities and integrate molecular investigations of stress memory mechanisms. Particular attention should be paid to the methodological requirements for detecting genuine lag effects and their ecological significance under climate change scenarios, including the potential for whole-canopy or ecosystem-level responses that extend beyond individual leaf measurements.

## Conclusion

This study revealed that *Acer campestre* exhibits threshold-dependent temporal regulation in response to thermal stress and critically highlights how sampling methodology influences the detection and interpretation of physiological lag effects. The 2022 daily measurements captured short-term thermal sensitivity under extreme heat, particularly in morning NPQ responses, whereas the 2023 weekly measurements generated correlations that reflected sampling structure rather than multi-day physiological memory.

The key methodological finding is that detecting genuine temporal dynamics requires sampling frequency aligned with the expected biological response timescale. Sparse sampling protocols can generate misleading correlations that appear to indicate physiological memory but are instead statistical artefacts. Thermal regime and sampling frequency were necessarily confounded in this study: daily sampling during the 2022 heatwave enabled resolution of fine-scale temporal responses, while weekly sampling in 2023 lacked the resolution to identify short-term dynamics.

For urban forest management, these insights emphasise that early-day photochemical sensitivity can serve as an informative indicator of heat stress, but reliable detection requires high-frequency measurements during heat events. Morning-targeted monitoring offers particular diagnostic value, as both years showed greatest thermal sensitivity in the early part of the day. Distinguishing true physiological processes from sampling-induced artefacts is therefore essential for designing effective stress-detection systems in urban environments.

Study limitations include the focus on a single species, differences in sampling protocols between years, and restriction to leaf-level fluorescence measurements. Future work should prioritise consistent high-frequency sampling across diverse thermal contexts, incorporate additional physiological and molecular indicators of heat stress, and extend assessments to multiple species, including non-native taxa commonly used in UK cities.

As climate variability intensifies, integrating temporal dynamics into urban forestry requires both understanding heat-response mechanisms and adopting monitoring strategies capable of detecting rapid physiological change. Failure to match sampling methodology to biological timescales risks misinterpreting artefactual correlations as true stress memory, with consequences for both scientific inference and practical decision-making.

### Author Contributions

Ramla Khan wrote the draft, collected the data, and performed the analysis as part of her PhD work. David Gowing and Philip Wheeler, in the position of supervisors, revised the document, gave suggestions for the analysis protocol, and data collection.

## Data Availability

The data that support the findings of this study are available at https://zenodo.org/records/17854643. It includes the raw OSP5+ data for the 2022 and 2023 seasons, weather data for 2022 and 2023 from the weather station on the Open University, MK, campus, and the fitted RLC curves of 2022.

## Abbreviations

SDGs: Sustainable Development Goals
NPQ: Non-Photochemical Quenching
ETR: Electron Transport Rate
GLMM: Generalised Linear Mixed Model
RLC: Rapid Light Curve
PAR: Photosynthetic Aperture Radar
CCF: Cross Correlation Function
